# Hemiptera Mitochondrial Control Region: New Sights into the Structural Organization, Phylogenetic Utility, and Roles of Tandem Repetitions of the Noncoding Segment

**DOI:** 10.3390/ijms19051292

**Published:** 2018-04-26

**Authors:** Kui Li, Ai-Ping Liang

**Affiliations:** 1 Key Laboratory of Zoological Systematics and Evolution, Institute of Zoology, Chinese Academy of Sciences, Beijing 100101, China; leekuy89@163.com; 2 College of Life Sciences, University of Chinese Academy of Sciences, Beijing 100049, China

**Keywords:** mitogenomes, control region, tandem replication, A+T content, Hemiptera

## Abstract

As a major noncoding fragment, the control region (CR) of mtDNA is responsible for the initiation of mitogenome transcription and replication. Several structural features of CR sequences have been reported in many insects. However, comprehensive analyses on the structural organization and phylogenetic utility, as well as the role of tandem replications (TRs) on length variation, high A+T content, and shift of base skew of CR sequences are poorly investigated in hemipteran insects. In this study, we conducted a series of comparative analyses, using 116 samples covering all 11 infraorders of the five currently recognized monophyletic groups in the Hemiptera. Several structural elements (mononucleotide stretches containing conserved sequence blocks (CSBs), TRs, and GA-rich region) were identified in the mitochondrial control region in hemipteran insects, without showing a consistent location. The presence and absence of certain specific structural elements in CR sequences show the various structural organizations of that segment among the five monophyletic groups, which indicates the diversification of the control region’s structural organization in Hemiptera. Among the many groups within Hemiptera, eight monophyletic groups and three consistent phylogenetic trees were recovered, using CSBs datasets by maximum likelihood and Bayesian methods, which suggests the possible utility of CR sequences for phylogenetic reconstruction in certain groups of Hemiptera. Statistical analyses showed that TRs may contribute to the length variation, high AT content, and the shift of base skewing of CR sequences toward high AT content in the Hemiptera. Our findings enrich the knowledge of structural organization, phylogenetic utility, and roles of tandem replication of hemipteran CR, and provide a possible framework for mitochondrial control region analyses in hemimetabolous insects.

## 1. Introduction

The control region (CR) is a requisite part of the mitogenome, which is also called an “A+T-rich region” for its extraordinarily high A+T content in insects [1]. Among the noncoding fragments in the mitogenome, the CR is a crucial segment, which is responsible for initiation of mtDNA transcription and replication [2,3,4]. This noncoding segment exhibits comparatively high rates of nucleotide substitution, dramatic divergence of primary nucleotide sequences, and remarkable variation in fragment length among species and individuals [5]. With the aforementioned properties, the CR has been considered as a kind of potential molecular marker to explore scientific issues of evolutionary biology from insects to mammals [5,6,7,8].

With extensive utilization of computational biology in molecular systematics, more and more mitogenomes were sequenced, both by traditional and next-generation sequencing in holometabolous and hemimetabolous insects, for phylogenetic and phylogeographic analyses. Moreover, just like mtDNA sequences, the CR has been consistently regarded as a prominent marker and widely applied for phylogenetic analyses in vertebrates [6,7,8]. However, in insects, CR sequences have often received little attention with respect to their dramatic nucleotide divergence and remarkable length variation among species and individuals, which become great obstacles for investigating the pattern of structural organization and use of phylogenetic reconstruction of this segment. As one of the important parts of mtDNA, several structural features have been reported in many insects. Adequate explorations of these structural features will provide a clear understanding of the CR’s structural organization and phylogenetic potential [5,6,7,8], which will also enrich our knowledge about mitogenome structural features and the use of phylogenetic reconstruction. Previous studies identified several conserved elements in Diptera, Lepidoptera, and Coleoptera, which were supposed to play an essential role in mtDNA replication in holometabolous insects [5,9,10]. Subsequently, relevant studies merely report some structural elements identified in CR sequences in several species, with little comparative analyses coupled with insect mitogenome research, which itself cannot reflect the general patterns of CR structural organization at the upper taxonomic levels [11,12]. A comparative analysis of this segment was conducted previously, mainly based on a limited number of holometabolous insects [5]. The analysis divided the CR into two groups. Group 1 contains a conserved region near the tRNA^Ile^ gene, and a variable domain including the remaining segment with high variation of the nucleotide sequence and length. While Group 2 did not contain distinct conserved or variable domains, some conserved sequence blocks (CSBs), tandem repetitions (TRs), and other structural elements were found scattered in the Group 2 control region sequences. Moreover, those conducting the study also believed that there was a possible utility for these structural elements in future phylogenetic studies. Thereafter, a few CSBs were also identified in many insects, such as Dipteran, Orthopteran, and Plecoptera [11,13,14]. However, no evidence has clearly confirmed that there are enough useful phylogenetic signals in insect CR sequences [11,15]. Most recently, phylogenetic analyses on bioluminescent Elateridae species proposed again that the CR might be used as a suitable marker for phylogenetic reconstruction [16]. However, these studies on the phylogenetic potential of CR just focus on holometabolous insects, and relevant research on hemimetabolous groups has not been reported up until now. It has been widely recognized that the occurrence of TRs are one of the most common events in CR sequences. Studies on roles of this structural element have revolved around the hypothesis that the variation of unit size and copy number is responsible for length variation of CR sequences among different species, and even individuals in the same species [5,17,18]. However, there was only limited data from holometabolous insects, which may be not applicable to hemimetabolous insects. Several analyses have shown that there are multiple TRs in CR sequences in insects [16,19], which suggests a distinct deficiency in evaluating the roles of TRs on length variation of CR sequences, simply based on unit size and copy number of the TR. Thus, in order to assess the role of TRs on length variation of CR sequences, the total TR length, synthesizing the information about the number, repeat unit size, and copy number variation of the structural element must be taken into account. In addition, it is still unknown whether the occurrence of TRs contributes to high AT content and base skewing of CR sequences toward high AT content. It has been widely recognized that CR sequences exhibit heavy base bias towards A and T [1]. The evaluation of TR’s roles on the AT content and base skewing of CR sequences toward high AT content will help us trace the potential molecular mechanism or evolutionary clues leading to this heavy base bias of CR sequences.

The order Hemiptera (Hem) has more than 82,000 described species, and exhibits the highest diversity among the hemimetabolous groups of insects [20]. Currently, Hemiptera comprises following five major monophyletic groups: Sternorrhyncha (Ste), Fulgoromorpha (Ful), Cicadomorpha (Cic), Heteroptera (Het), and Coleorrhyncha (Col) [21,22]. Heteroptera also can be subdivided into seven infraorders: Enicocephalomorpha (Eni), Dipsocoromorpha (Dip), Gerromorpha (Ger), Nepomorpha (Nep), Leptopodomorpha (Lep), Cimicomorpha (Cim), and Pentatomomorpha (Pen) [23]. During the evolutionary process, hemipteran insects exhibit numerous morphological, behavioral, and physiological diversities among different groups, and even within the same group [24,25,26,27]. Therefore, Hemiptera makes an ideal subject among hemimetabolous groups for comprehensive analyses of the mtDNA control region. The investigation of structural features and phylogenetic potential of hemipteran CR sequences will help us to not only draw the patterns of CR structural organization and usability of CR for phylogenetic reconstruction in different groups, but also increase our knowledge of the structure characteristics and phylogenetic utility of hemipteran mtDNA sequences. Although a few structural elements (e.g., TRs, Poly(T), and stem-loop structures) of CR sequences have been elucidated with mitogenome analyses in the Hemiptera, these features only occur in some species [12]. Thus, these structural elements cannot clearly reflect general patterns of the structural organization of mtDNA control regions in the Hemiptera. In this study, we conducted a series of comparative analyses on hemipteran CRs for the overall pattern of structural organization and phylogenetic potential, and roles of TRs in the length variation, high AT content, and base skewing toward high AT content in mtDNA control regions in different groups of the Hemiptera.

## 2. Results

### 2.1. The Structural Organization

**Figure 1:**
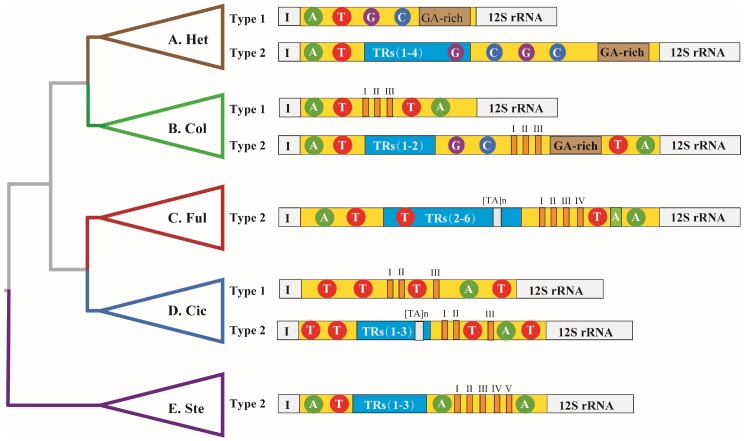
The structural organization pattern of the control region (CR) in Hemiptera groups. The CR is presented with a yellow long frame. Nucleotide stretches are shown in circles with different colors. The long A stretch is exhibited with a grass green bar; GA-rich regions are represented by brown bars; tandem replications (TRs) are shown by light blue bars with the different numbers; CSBs are shown by orange bars. TA is represented by white bar. Phylogenies of Hemiptera were used from the latest study [28]. Abbreviation: (**A**) Het: Heteroptera; (**B**) Col: Coleorrhyncha; (**C**) Ful: Fulgoromorpha; (**D**) Cic: Cicadomorpha; (**E**) Ste: Sternorrhyncha.

In total, 116 CR sequences were retrieved from the hemipteran mitochondrial genomes published in NCBI (https://www.ncbi.nlm.nih.gov/), which covers all 11 infraorders of the five currently recognized monophyletic groups in Hemiptera. Results showed that no distinctly conserved or variable domains were identified in the mtDNA CR sequences within the Hemiptera (Figure 1). In spite of this, several structural elements were found to be randomly scattered in CR sequences with different locations among species of the Hemiptera (Figure 1A–E and Table S1). A few short A or T stretches, about 6–10 base pairs (bp) in length, were scattered in CR sequences without uniform order in almost all species throughout different infraorders. One long A stretch, about 23 bp, was identified near the 3′ terminal of CR sequences in almost all species of Fulgoromorpha (Figure 1C), while short G or C stretches of about 6–10 bp in CR sequences were identified in Heteroptera and Coleorrhyncha. Meanwhile, a conserved GA-rich region was also discovered in the 3′ terminal of CR sequences close to the 12S rRNA in most species of Heteroptera and Coleorrhyncha. Besides, several short CSBs were identified in CR sequences in eight infraorders, two super families, and nine families, mainly by Multiple alignment program MAFFT method (Figure 1 and Figure S1). Just like the short mononucleotide stretches, these CSBs were also found to scattered in CR sequences without a consistently fixed location within these groups. At the infraorder level, there were no CSBs in CR sequences of Gerromorpha and Pentatomomorpha. Additionally, Leptopodomorpha was excluded for CSB identification, due to only one CR sequence being published in the database. Among the eight infraorders with CSBs in their CR sequences, Sternorrhyncha (I~V) and Dipsocoromorpha (I and II) exhibited the most and the least CSBs, respectively. At the superfamily level, four CSBs total were identified in Lygaeoidea (I~III) and Pentatomiodea (I). Within the nine families, CR sequences of family Aphididae (I~VI) and Miridae (I) contained the most and the least CSBs, respectively. In addition, TRs with different lengths were also identified in CR sequences in almost all species among the five monophyletic groups (Figure 1 and Table S1). More than one TR appeared in CR sequences in the majority of species. Compared with other groups, CR sequences in Fulgoromorpha contained up to six TRs. Besides, some G or C stretches were presented in a few TR sequences of certain species of Heteroptera. (TA)n stretches were also identified in TR sequences in both Fulgoromorpha and Cicadomorpha. Also, no TR was observed in some CR sequences, mainly in Heteroptera species, except Sternorrhyncha and Fulgoromorpha (Table S1). Additionally, we failed to identify stem-loop structures in hemipteran CR sequences.

### 2.2. Phylogenetic Reconstruction

**Figure 2:**
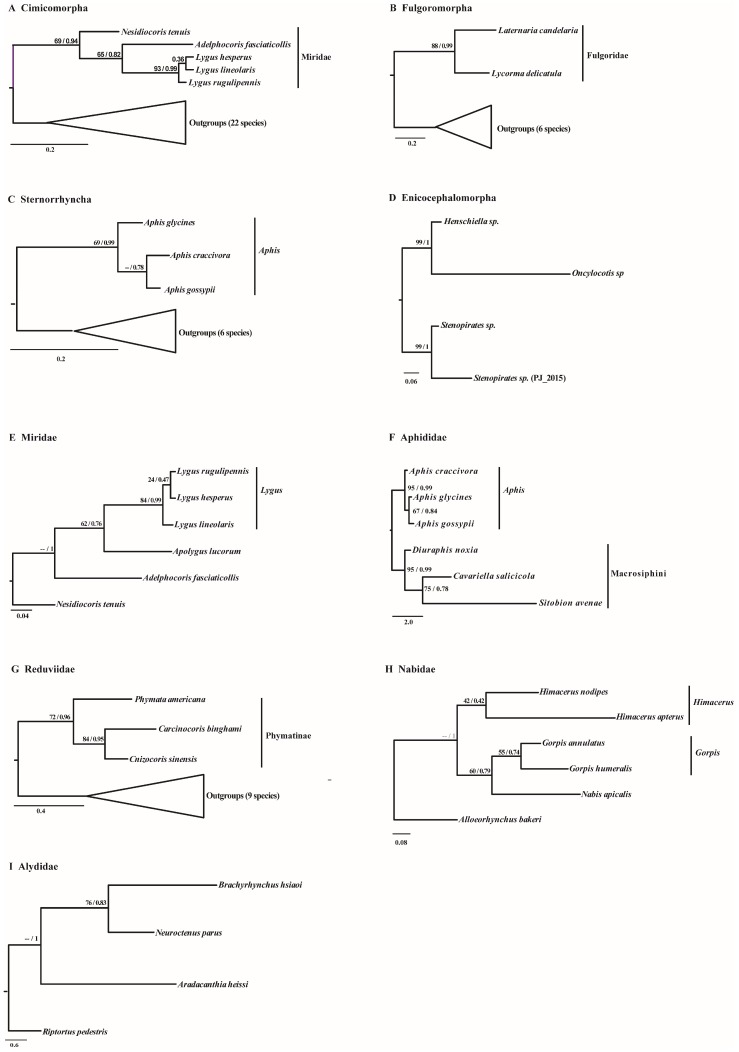
Phylogenetic trees (**A**–**I**) of different groups in Hemiptera, based on 19 conserved sequence block (CSB) datasets recovered by Bayesian and maximum likelihood analyses. Maximum likelihood bootstrap values and Bayesian posterior probability are indicated at each node.

CSBs of CR sequences detected in 19 groups were used to evaluate the potential of CR sequences for phylogenetic reconstruction. Results showed that the best-fit models were F81, GTR+G, HKY, and HKY+G for these CSBs among groups (Table 1). Phylogenetic reconstruction based on CSB datasets identified at the infraorder level showed that the monophyly of family Miridae and Fulgoridae was well-supported with high bootstrap support (BS) by Bayesian (BS > 0.90) analyses (Figure 2A,B). The result was also verified by maximum likelihood (ML) analyses, although support values were slightly lower than Bayesian analyses (Figure 2A,B). Simultaneously, genus *Lygus* (BS = 93/0.99) and Aphis (BS = 69/0.99) were also well supported as monophyletic groups by both methods, although the support value of ML analyses was relatively low (Figure 2A,C). In addition, a dataset with four species in infraorder Enicocephalomorpha recovered a highly-supported tree with a BS of 99/1 in each node (Figure 2D). At the family level, phylogenetic analyses were also conducted using CSBs of CR sequences identified by ML and Bayesian methods. Just like the case at the infraorder level, results showed that the monophyly of genus Lygus (BS = 84/0.99) and Aphis (BS = 95/0.99) were also well-supported with high bootstrap support (Figure 2E,F). Meanwhile, the monophyly of tribe Macrosiphini (Aphididae) (BS = 95/0.99) and subfamily Phymatinae (Reduviidae) (BS = 72/0.96) was well-recovered (Figure 2F,G). Although the bootstrap support was not high, genus Himacerus and Gorpis were also recovered as monophyletic groups by both methods (Figure 2H). Besides, datasets formed by CSBs detected in families Miridae and Alydidae also recovered well-supported trees, with high bootstrap support in each node by both methods (Figure 2E,I). In other groups, consistent phylogenetic trees with monophyletic subgroups and high bootstrap support could not be recovered in our analyses based on CSBs datasets.

### 2.3. Roles of Tandem Replicationss on Length Variation of Control Region Sequences

**Figure 3:**
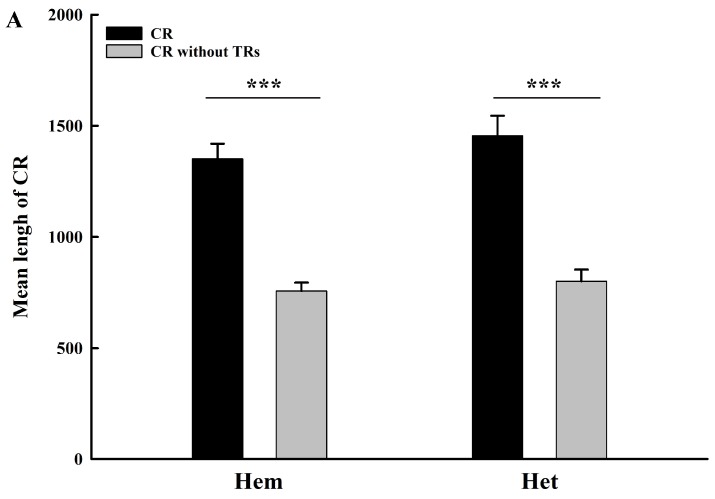

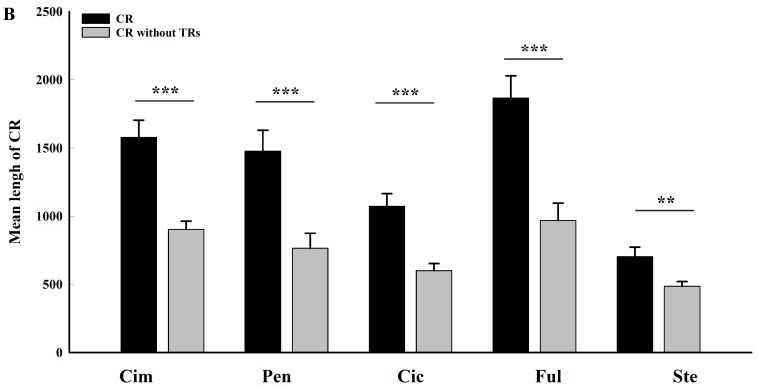
The variation of length between CRs and the remaining part without tandem replications (TRs) in different hemipteran groups (**A**,**B**). Data is displayed as mean ± SE (Standard Error). The significance level is *p* < 0.05. Two asterisks: *p* < 0.01; Three asterisks: *p* < 0.001. Abbreviation: Hem: Hemiptera; Het: Heteroptera; Cim: Cimicomorpha; Pen: Pentatomomorpha; Cic: Cicadomorpha; Ful: Fulgoromorpha; Ste: Sternorrhyncha.

**Figure 4:**
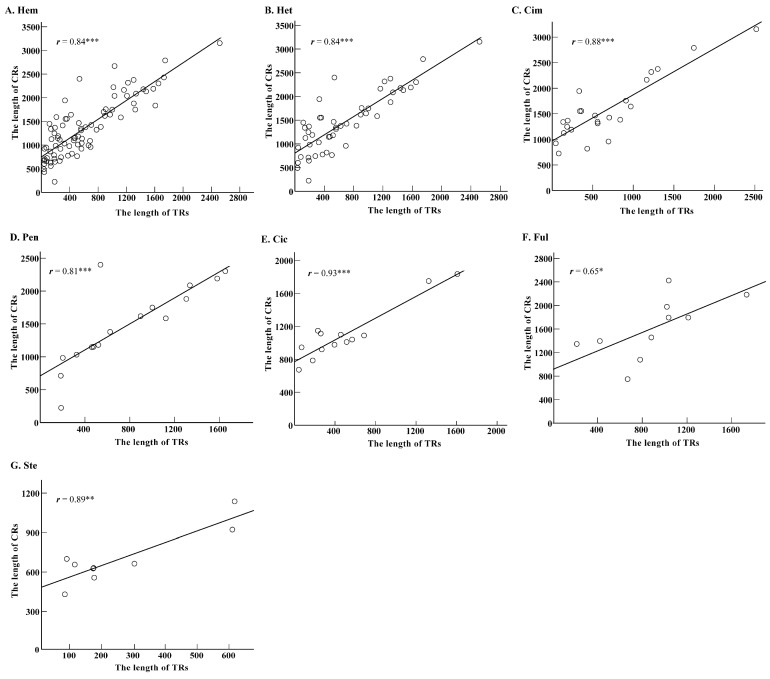
The correlation of length between TRs and CRs in Hemiptera groups (**A**–**G**). The significance level is *p* < 0.05 with one asterisk. Two asterisks: *p* < 0.01; Three asterisks: *p* < 0.001. The abbreviation of different groups was referred in Figure 1 and Figure 3.

Results showed that the length of hemipteran CR sequences was about 220~3155 bp among sampled species (Table S1). The length of most CR sequences ranged from 500~2500 bp, and exhibited an obvious length variation among species in Hemiptera. The species with the longest (*Kleidocerys resedae*: 220 bp) and the shortest (*Nesidiocoris tenuis*: 3155 bp) CR sequence in mtDNA belong to infraorders Pentatomomorpha and Cimicomorpha, respectively. In addition, the length of TRs (35~2517 bp) and remain sequences (37~1991 bp) without TRs also exhibit obvious length variations (Table S1). Statistical analysis showed that there was a significant difference in length between the full CR sequences and the remaining part without TRs, with *p* < 0.001 for all of Hemiptera and suborder Heteroptera (Figure 3A). Simultaneously, similar results were also verified in infraorders Cimicomorpha, Pentatomomorpha, Cicadomorpha, Fulgoromorpha, and Sternorrhyncha, with *p* < 0.001 (Figure 3B). These results suggest a marked role of TRs on length increase of hemipteran CR sequences. Correlation analyses showed that there was a remarkable positive correlation between the total length of TRs and that of CR sequences in the whole Hemiptera and suborder Heteroptera, with relatively high correlation coefficient (*r*) greater than 0.8 and *p* < 0.001 (Figure 4A,B). Similar analysis on infraorders also exhibited the same results, with relatively high *r* and *p* < 0.05 (Figure 4C,G).

### 2.4. Roles of Tandem Replicationss on A+T Content and Base Skewing of Control Region Sequences

**Figure 5:**
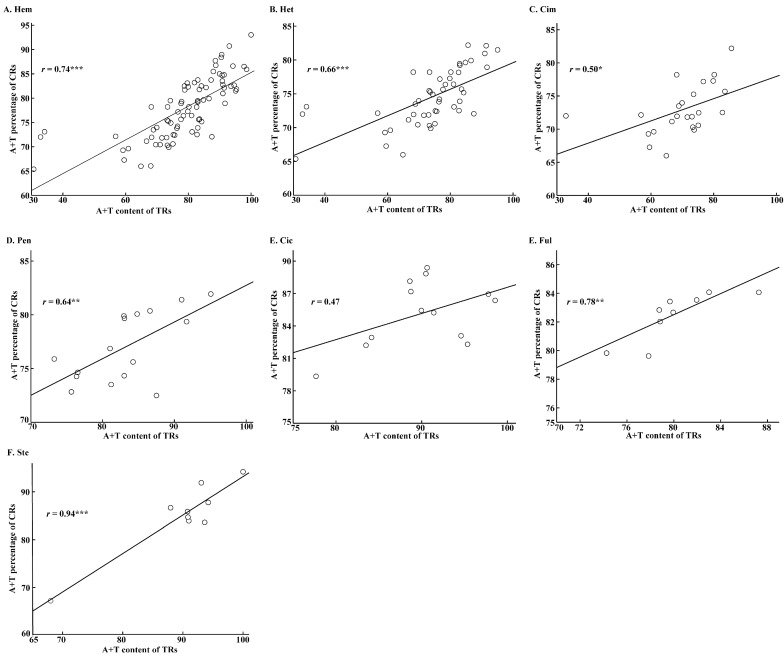
The correlation of A+T content between TRs and CRs in different groups (**A**–**F**) of Hemiptera. The significance level is *p* < 0.05 with one asterisk. Two asterisks: *p* < 0.01; Three asterisks: *p* < 0.001. The abbreviation of different groups was referred in Figure 1 and Figure 3.

The AT content of CR sequences of the Hemiptera insects ranged from 63.87% to 93.02% (Table S1), which is a relatively high proportion of the base composition. The CR sequence in *Sitobion avenae* (Sternorrhyncha) presents the highest AT content (93.02%). By contrast, the CR sequence in *Lethocerus deyrollei* (Nepomorpha) shows the lowest AT content (63.87%). Besides, TR sequences exhibit high AT percentage in almost all Hemiptera insects, especially in Sternorrhyncha, where the AT content is 100% (Table S1). Correlation analyses show remarkable positive correlation of AT content between TR and CR sequences in the whole Hemiptera and suborder Heteroptera, with *r* > 0.65 and *p* < 0.001 (Figure 5A,B). Similar analyses present the same case in infraorders Cimicomorpha, Pentatomomorpha, Fulgoromorpha, and Sternorrhyncha, with *r* > 0.5 and *p* < 0.05, except Cicadomorpha (Figure 5C–F). In addition, results also showed that CR sequences usually exhibited remarkable T- or G-skewing in most infraorders, while Dipsocoromorpha (C-skew), Gerromorpha (A-skew), and Nepomorpha (A-skew) usually exhibited obviously different skews (Table 2 and Table S1). Besides, infraorder Cimicomorpha/Sternorrhyncha and Enicocephalomorpha/Fulgoromorpha exhibited inconspicuous AT- and GC-skews, respectively (Table 2 and Table S1). When TRs were deleted, the skewed pattern was not dramatically affected; however, the shift of the CR base skew still occurred in a few species (Table 3). Among the shift of the skew pattern, infraorder Pentatomomorpha usually exhibited a shift towards an A-skew, while Cicadomorpha usually exhibited a shift towards T-skew. By comparison, Cimadomorpha and Fulgoromorpha usually exhibited a shift towards a G-skew. Besides, Cimicomorpha also exhibited some shift toward an A- or T-skew.

## 3. Discussion

With rapid development of sequencing technologies, more and more CR sequences have been published with mitochondrial genome analyses in insects [12,29]. The boom in this kind of data provides us ideal materials to evaluate the structural features and roles of TRs on length variation and the base composition of CR sequences among groups in hemipteran insects. A better understanding of these parameters will provide deep insight into the general patterns of structural organization, phylogenetic utility, and potential molecular mechanisms or evolutionary clues leading to high AT content in CR sequences. 

### 3.1. The Conservation and Diversification of Control Regions in Hemiptera

Our analyses reveal that CR sequences consist of several structural elements (mononucleotide stretches, CSBs, TRs and GA-rich regions) without obvious conserved and variable domains in Hemiptera insects (Figure 1), which implies that the CR of hemipteran insects belongs to the Group 2 control region, as described previously [5]. We also have found that there are no TRs in CR sequences in some species (Figure 1). Previously, CRs of the fly *Haematobia irritans* were classified into three types, based on length variation caused by TRs [19]. When it comes to the occurrence of TRs, the CRs of Hemiptera insects also can be roughly divided into two types: type 1, without a TR; and type 2 with TRs. Unfortunately, we failed to find stem-loop structures in CR sequences in the Hemiptera. Earlier studies have discovered conserved motifs at the flanking sequence of stem-loop structures, which may be used to define the secondary structure [30]. Indeed, these conserved motifs were also identified at the flanking sequence of the secondary structure in stoneflies [13]. We could not find these motifs on hemipteran CR sequences, which obstructed the identification of an accurate location for the secondary structure. However, the secondary structure has been identified in hemipteran CR sequences in certain species [12]. We also failed to find the homologous fragment of these stem-loop structures in other hemipteran CR sequences, due to great divergence of primary sequences of the noncoding segment. Analyses of CR sequences on Muscidae flies have shown that the primary nucleotide sequence and relative position of the secondary structure are not conserved [19]. The secondary structure in hemipteran CR sequences may also exhibit a similar case, as observed in Muscidae flies, which increases the difficulty of identifying the stem-loop structures. Thus, effective methods need to be developed for the identification of the structural element in Hemiptera.

Wang et al. have summarized several structural elements of CR sequences in hemipteran insects, based on previous studies, along with several mitochondrial genome analyses [12]. Yet some of these elements are only presented in the minority of species, which cannot clearly reflect the general structural organization of CR sequences in Hemiptera. Our results revealed that short A and T stretches may be the common feature, due to the stretches’ frequent occurrence in almost all CR sequences in hemipteran insects. In contrast, short G and C stretches and the GA-rich region only appear in Heteroptera and Coleorrhyncha CR sequences. The lack of TRs in CR sequences in Gerromorpha and Pentatomomorpha causes an obvious difference in CR structural organization between Heteroptera and Coleorrhyncha. Although there are several similar structural features among Cicadomorpha, Fulgoromorpha, and Sternorrhyncha, the occurrence of (TA)n in TRs of CR sequences in almost all species of Cicadomorpha and Fulgoromorpha may be a distinct trait of the segment, in order to distinguish the two groups against Sternorrhyncha. Moreover, the long A stretches (~23 bp) of CR sequences, only detected in all Fulgoromorpha, can also be used as a discriminating CR feature between this infraorder and Cicadomorpha. Therefore, the presence and absence of these specific structural elements show different patterns of CR structural organization among the five monophyletic groups, which may suggest the diversification of structural organization of CR sequences in the Hemiptera. For low levels of primary sequence similarity, it is difficult to investigate the conservation of CR sequences of insects. A previous study hypothesized that the conservation of CR can be indicated by the occurrence of location, order, and overall distribution pattern of structural elements [5]. The proposition has been validated in some groups of Diptera and Coleoptera with consistent structural features, respectively [14,16,19]. The diverse structural organization of hemipteran CR sequences indicates that the structure is not always conserved, as previously reported in other groups, which may also hint at the differences of CR structural organization between holometabolous and hemimetabolous insects. As the most biodiverse group in hemimetabolous insects, the Hemiptera presents a complex evolutionary pattern, with various habitats and morphological characteristics [20,31]. The diversification of CR structural organization may be a reflection at molecular scale of hemipteran biodiversity.

### 3.2. The Potential Phylogenetic Utility of Control Regions in Hemiptera

Phylogenetic reconstruction, based on 19 datasets formed by CSBs, showed that the monophyly of family Miridae and Fulgoridae, subfamily Phymatinae, tribe Macrosiphini, and genus *Lygus*, *Aphis*, *Himacerus*, and *Gorpis* were recovered by both Bayesian and ML analyses, with comparatively high bootstrap support. The monophyly of these groups was also recovered by previous studies [28,32,33,34,35], which hints at the underlying utility of CR for phylogenetic reconstruction. Furthermore, just as in a previous study by mitogenome analyses [28], well-supported trees were recovered in infraorder Enicocephalomorpha and families Alydidae and Miridae, which further suggested the possibility of CR for phylogenetic reconstruction. For conservation in both structural and primary sequences of the CR in vertebrates, it has been widely used as an ideal molecular marker for phylogenetic, phylogeography, and population genetic analyses [6,7,8]. Although it can be applied in population analyses [11], the potential of CRs for phylogenetic usage with interspecific or higher levels of insects has been debated for decades. Phylogenetic analyses on the genus *Jalmenus* (Lepidoptera) showed little functional phylogenetic signal in CR sequences [15]. However, a subsequent study proposed that highly-conserved domains with reasonable size still can be used for phylogenetic reconstruction [5]. Recently, reanalysis of CRs on Elateridae bioluminescent groups has confirmed that the conserved domain may be suitable for phylogenetic reconstruction [16]. The eight monophyletic groups, from the genus to family level, and three well-supported trees recovered in our study also support that the CR may be a suitable molecular marker for phylogenetic analyses. However, the monophyletic groups and well-supported trees recovered in our study are still relatively few among the numerous groups within Hemiptera, which may imply a limited potential of CR for extensive application of phylogenetic analyses within the order. Therefore, the CR may be merely used in a few groups, as mentioned above, and cannot be widely applied for phylogenetic reconstruction in the Hemiptera. Compared with other parts of mtDNA, the usage of the CR has been nearly abandoned in insect phylogenetic analyses, with respect to its great divergence of the primary sequence. Although CR sequences exhibit limited phylogenetic utility, as reported in our study, results still indicate that the CR can be used for phylogenetic analyses at least in some hemipteran groups. Besides, the phylogenetic utility of CR sequences also enriches use efficiency of mtDNA sequences in insect phylogenetic reconstruction. Generally, short molecular markers usually contain fewer informative sites for phylogenetic reconstruction, which may cause low utility for phylogenetic analyses. This may be the reason why we failed to recover consistent phylogenetic trees with monophyletic subgroups and high support in other groups. In spite of many obstacles, such as great divergence of primary sequence, difficultly in alignment, and short conserved domains without enough informative sites, CR sequences usually evolve faster than other genes in the mitochondrial genome [5]. If a CR sequence can provide enough informative sites, it still can be used with other molecular markers for a better phylogenetic tree. 

### 3.3. Roles of Tandem Replications on Length Variation, AT Content, and Shift of Base Skewing in Hemiptera

Obvious length variation was observed in CR sequences in the Hemiptera, from 220 bp (*Kleidocerys resedae*) to 3155 bp (*Nesidiocoris tenuis*) (Table S1). Previous studies, mainly based on limited data from holometabolous insects, have revealed that the size and copy number of the repeat unit are responsible for the length variation of CR sequences among species, and even in individuals [5,17,18]. We found that more than one TR was identified in CR sequences, which implied that it is not enough to evaluate the role of TRs on length variation of CR sequences simply based on the unit size and copy number of the TR in the Hemiptera. Thus, when evaluating the role of TRs on the length variation of CR sequences in Hemiptera, the total length of TRs containing integrative information about the number, copy number, and size of different repeat units of the structural element must be taken into consideration. Our results reveal that TRs and the remaining segments also exhibit length variation. Moreover, TRs with such length variation lead to a remarkable length increase of CR sequences in Hemiptera (Figure 3). Length variation of the remaining segments may also contribute to a CR’s length variation, which intuitively seems to obstruct our evaluation of TR roles on length variation of CR sequences. Nevertheless, in consideration of the prominent impact of TRs on length increase of CR sequences, the role of TRs on the variation of length in CRs still can be deduced, as long as there is a remarkable positive correlation of length variation between TR and CR sequences. Indeed, the remarkable positive correlation really exists in all tested taxonomical levels (Figure 4). Therefore, we hypothesize that TRs with variable lengths among species may contribute to the length variation of CRs in Hemiptera.

Interestingly, just like the case in CR sequences, high AT content was also detected in TR sequences. Despite not occurring in all infraorders, a remarkable positive correlation of AT content between TR and CR sequences was observed in some infraorders (Figure 5), which implies that high AT content of TR sequences may contribute to the high proportion of AT content of CR sequences in the Hemiptera. We also found that T- and G-skews in CRs are very common in Hemiptera. Taking the complementarity of the N- and J-strand into consideration, the skew pattern is consistent with most hemipteran mitogenomes [12]. When TRs were removed, the CR sequence in some species exhibited A- or T-skewing (Table 3 and Table S1), which suggests a possible role of TRs on base composition of the noncoding segment in hemipteran insects. The roles of TRs on base composition also hint at a potential impact of TRs on high AT content of CR sequences. Thus, TRs with a high AT percentage possibly contribute to the high AT content of CR sequences, which may provide a potential molecular mechanism or evolutionary clue leading to the heavy base bias in the noncoding segment. The reasons for high AT content of CR sequences in insects have not been investigated or surveyed until now. A previous study hypothesized that mutation pressure towards A/T may have caused the high AT content of CR sequences in mtDNA [5]. When TRs were deleted, the remaining part of the CR also showed high AT content, which seem to support the speculation. Therefore, we speculate that both the mutation towards A or T base and the occurrence of TRs with high percentages of AT bases may contribute to the overall high A+T content of CR sequences in the Hemiptera. 

## 4. Materials and Methods 

### 4.1. Sequence Retrieval, Alignment, and Structural Analysis

CR sequences of hemipteran insect were retrieved from the National Center for Biotechnology Information (NCBI: http://www.ncbi.nlm.nih.gov/), according to the GenBank accession numbers of mitogenomes provided in previous studies [28,36]. We also searched newly-published data from NCBI for a wide range of sample coverage. In order to avoid the random matching, sequence alignment of hemipteran CRs was conducted using MAFFT, BioEdit 7.0, and MEGA 6.0 [37,38,39]. The CSBs were identified mainly by MAFFT, with the assistance of multi-purpose tools BioEdit 7.0 and MEGA 6.0. An online Tandem Repeats Finder program was used to detect the TRs of CR sequences in each species of Hemiptera [40]. The location and display of tandem repeat units in DNA sequences were also identified by this program. When multiple reports of a repeat at different pattern sizes was encountered, the largest pattern size was counted, to improve alignment [40]. In addition, the identification of the stem-loop structure was also surveyed using Mfold [41]. Given that the stem-loop structures were identified in certain hemipteran species [12], primary sequences of the secondary structures were also used as templates to identify the homologous fragment in other hemipteran CR sequences.

### 4.2. Phylogenetic Reconstruction

CSBs detected in different groups were used in phylogenetic analyses, to evaluate the potential of CR sequences for phylogenetic application. When multiple CSBs were encountered in a group, the combined data were used as a dataset, to obtain a higher number of informative sites. The best-fit model of nucleotide substitution was tested using Bayesian information criteria (BIC) by jModelTest 2.1.7 [42]. Bayesian inference (BI) analyses were performed by MrBayes version 3.2.6 under the following parameters: four runs of 100,000 generations and 25% burn-in [43]. Stationarity was considered to be reached when the average standard deviation of split frequencies was less than 0.01. The posterior probability (PP) of each node was used to estimate tree support. Meanwhile, maximum likelihood (ML) analyses were also performed, under the optimal models calculated by jModelTest 2.1.7, using software PhyML v 3.0 [44]. The nodal support of branches was evaluated by bootstrap analysis with 1000 replicates. 

### 4.3. Statistical Analyses

In order to evaluate roles of TRs on length variation, the AT content and the shift toward an AT- or GC-skew (AT-skew: A-T/A+T; GC-skew: G-C/G+C) of CR sequences in Hemiptera insects, SPSS 17.0 was employed for all statistical tests [45]. In order to avoid the inaccuracy of statistical results, infraorders less than seven samples (Coleorrhyncha, Enicocephalomorpha, Dipsocoromorpha, Gerromorpha, Leptopodomorpha, and Nepomorpha) were excluded from statistical analyses. Detailed statistical information were summarized in Table S1. The normal distribution of the total length, AT content, and length without TRs of CR sequences was tested by Kolmogorov–Smirnov test [46]. Comparative analysis was conducted by Student’s *t*-tests with a significance level of 0.05. The Pearson correlation analysis was also used for statistical analyses.

## 5. Conclusions

In the present study, 116 CRs of hemipteran mtDNA were retrieved. The species sampled in the study represented all 11 infraorders of the five monophyletic groups currently recognized in the Hemiptera. Comparative comprehensive analyses were conducted for the general structural organization, phylogenetic utility, and roles of TRs on length variation, high AT content, and AT/GC skewing. Our results have shown that the structural organization of CR sequences have diversified, due to the presence and absence of certain specific structural elements among the five monophyletic groups (Sternorrhyncha, Fulgoromorpha, Cicadomorpha, Heteroptera and Coleorrhyncha). The diversity of structural organization of hemipteran CR sequences refreshes the current knowledge that there are different patterns of CR structural organization among groups of insects. In addition, the diversified patterns of structural organization may be a reflection of the molecular scale of biodiversity of Hemiptera. Eight monophyletic groups and two consistent phylogenetic trees have been recovered among numerous groups in the Hemiptera, which suggests that the CR may be suitable for phylogenetic utility in some groups of Hemiptera. As part of mtDNA, the phylogenetic utility of CR will increase the efficiency of the use of mtDNA sequences in hemipteran phylogenetic analyses. Besides, we also have found that TRs may contribute to length variation of CR sequences, high AT content, and the shift of base skewing toward high AT content, which confirms previous research [5]. These results mentioned above enrich the knowledge of CR sequences with regards to the pattern of structural organization, phylogenetic utility, and roles of TR, which also promotes the understanding of mtDNA organizational features and phylogenetic usage. As a group of hemimetabolous insects with the highest diversity [20], our findings also provide a possible framework for CR analyses of hemimetabolous insects. However, we still don’t clearly know the detailed molecular mechanisms behind the high AT content, and there are no reliable methods for stem-loop identification in CR sequences. Therefore, a better understanding of these issues should be well evaluated in future studies.

## Figures and Tables

**Table 1:** The best-fit models among groups.

Groups		Best-Fit Model	−lnL	BIC
Infraorder level	Cicadomorpha	HKY+G	840.293	1883.474
	Cimicomorpha	HKY+G	745.3419	1700.95
	Coleorrhyncha	HKY	227.3687	490.2786
	Dipsocoromorpha	HKY	160.6696	352.9492
	Enicocephalomorpha	F81	251.9445	543.0995
	Fulgoromorpha	HKY	330.6916	745.6133
	Nepomorpha	HKY	111.504	264.2301
	Sternorrhyncha	HKY+G	811.118	1721.88
Family level	Alydidae	HKY	197.9267	434.1398
	Aphididae	HKY+G	792.3208	1664.487
	Cercopidae	HKY	258.9371	586.3942
	Cicadellidae	HKY	437.4311	964.5663
	Cicadidae	F81	79.49639	182.3182
	Delphacidae	F81	440.9577	937.0325
	Lygaeoidea	F81	216.8583	464.3028
	Miridae	F81	188.896	428.9057
	Nabidae	HKY+G	807.0794	1689.418
	Pentatomiodea	HKY	294.1281	669.885
	Reduviidae	GTR+G	1281.578	2712.585

BIC means the bayesian information criteria.

**Table 2:** Skew patterns among infraorders in the Hemiptera.

Infraorder	A-Skew	T-Skew	G-Skew	C-Skew
Cim	--	--	√	--
Dip	--	√	--	√
Eni	--	√	--	--
Ger	√	--	√	--
Nep	√	--	√	--
Pen	--	√	√	--
Col	--	√	√	--
Cic	--	√	√	--
Ful	--	√	--	--
Ste	--	--	√	--

The symbol “–” means no corresponding skew, “√” means corresponding skew.

**Table 3:** The shift to an AT- or GC-skew of CRs in Hemiptera.

Infraorder	Species	AT-Skew	AT-Skew ^a^	GC-Skew	GC-Skew ^b^
Cim	*Cimex lectularius*	0.01	−0.08	−0.14	0.40
Cim	*Corythucha ciliata*	--	--	−0.1	0.01
Cim	*Gorpis annulatus*	--	--	−0.02	0.10
Cim	*Gorpis humeralis*	0.01	0.00	0	0.09
Cim	*Himacerus apterus*	--	--	−0.04	0.12
Cim	*Himacerus nodipes*	--	--	−0.04	0.00
Cim	*Lygus hesperus*	−0.07	0.04	--	--
Cim	*Lygus lineolaris*	−0.01	0.04	--	--
Cim	*Nesidiocoris tenuis*	−0.01	0.09	--	--
Cim	*Scotomedes* sp.	0.04	−0.08	--	--
Pen	*Aradacanthia heissi*	−0.03	0.06	--	--
Pen	*Dalcantha dilatata*	−0.01	0.00	--	--
Pen	*Dolycoris baccarum*	−0.06	0.00	--	--
Pen	*Dysdercus cingulatus*	−0.18	0.04	--	--
Pen	*Nezara viridula*	−0.09	0.03	--	--
Pen	*Sastragala edessoides*	0.02	−0.01	--	--
Pen	*Urochela quadrinotata*	0.03	−0.01	--	--
Cic	*Callitettix braconoides*	--	--	−0.04	0.07
Cic	*Empoasca vitis*	0.01	−0.01	--	--
Cic	*Homalodisca vitripennis*	--	--	0.01	−0.02
Cic	*Leptobelus* sp.	0.01	−0.02	--	--
Cic	*Yanocephalus yanonis*	0.01	−0.05	--	--
Ful	*Nilaparvata lugens*	--	--	−0.17	0.03
Ful	*Sogatella furcifera*	0.00	0.05	−0.11	0.10
Ste	*Aleurocanthus spiniferus*	0.05	−0.15	−0.16	0.02

Negative sign indicates a T- or C-skew, while others are an A- or G-skew. ^a^ AT-skews have been evaluated after deleting TRs; ^b^ GC-skews have been evaluated after deleting TRs.

## Supplementary Materials

Supplementary materials can be found at http://www.mdpi.com/1422-0067/19/5/1292/s1.

## Abbreviations

Cic	Cicadomorpha
Cim	Cimicomorpha
Col	Coleorrhyncha
CR	Control region
CSB	Conserved sequence block
Dip	Dipsocoromorpha
Eni	Enicocephalomorpha
Ful	Fulgoromorpha
Ger	Gerromorpha
Hem	Hemiptera
Het	Heteroptera
Lep	Leptopodomorpha
Pen	Pentatomomorpha
TR	Tandem repetition
