# Supplementary material for: Hemiptera Mitochondrial Control Region: New Sights into the Structural Organization, Phylogenetic Utility, and Roles of Tandem Repetitions of the Noncoding Segment
Source: Int J Mol Sci. 2018 Apr 26;19(5):1292. doi: 10.3390/ijms19051292 (PMC5983824; doi:10.3390/ijms19051292)
Supplement: Supplementary file 1 [file Figure S1.pdf › ijms-270316-supplementary material/Figure S1.pdf]

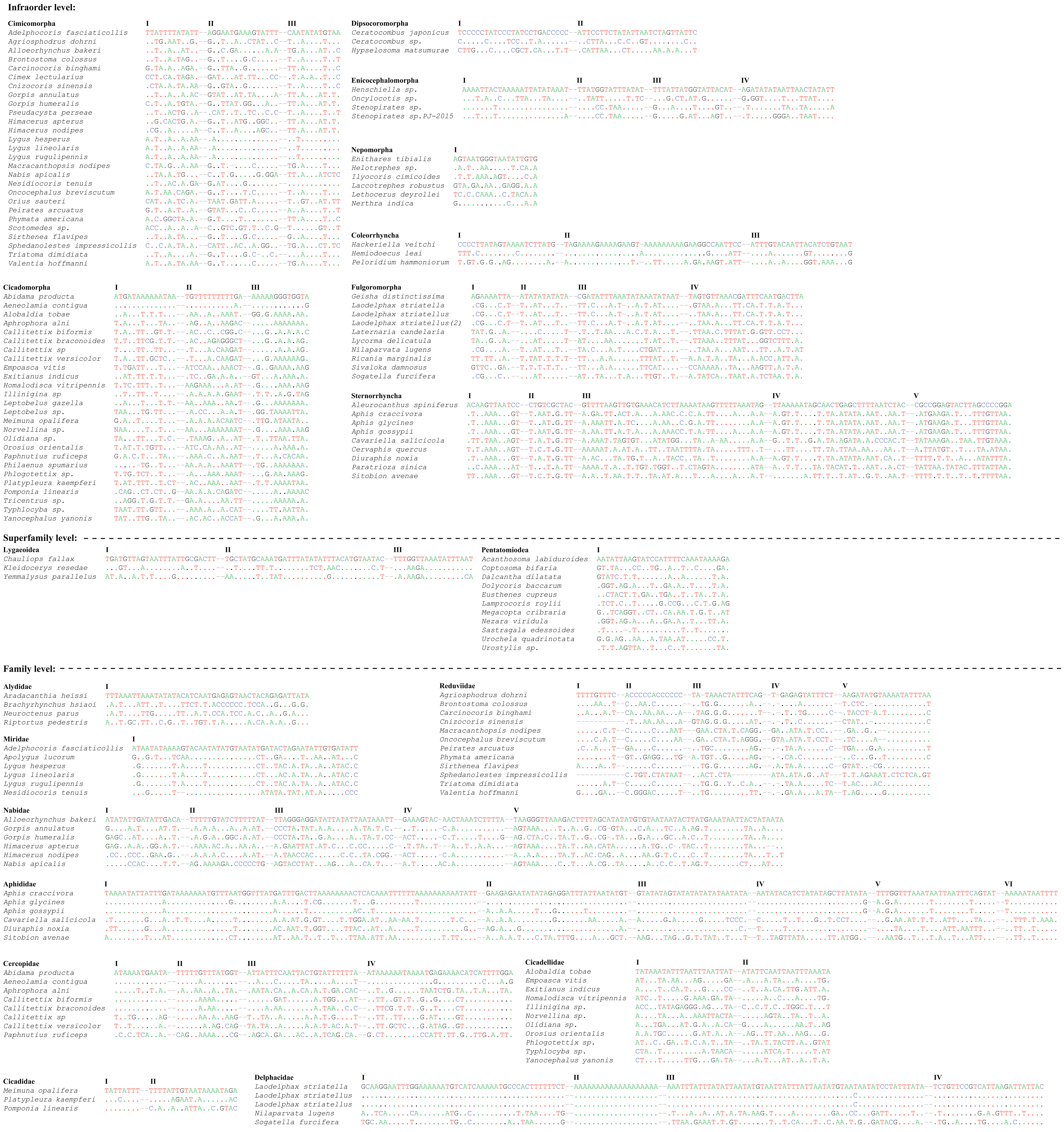
Infraorder level:
Cimicomorpha
Adelphocoris fasciaticollis
Agriosphodrus dohrni
Alloeorhynchus bakeri
Brontostoma colossus
Carcinocoris binghami
Cimex lectularius
Cnizocoris sinensis
Gorpis annulatus
Gorpis humeralis
Pseudacysta perseae
Himacerus apterus
Himacerus nodipes
Lygus hesperus
Lygus lineolaris
Lygus rugulipennis
Macracanthopsis nodipes
Nabis apicalis
Nesidiocoris tenuis
Oncocephalus breviscutum
Orius sauteri
Peirates arcuatus
Phymata americana
Scotomedes sp.
Sirthenea flavipes
Sphedanolestes impressicollis
Triatoma dimidiata
Valentia hoffmanni
Dipsocoromorpha
Ceratocombus japonicus
Ceratocombus sp.
Hypselosoma matsumurae
Enicocephalomorpha
Henschiella sp.
Oncylocotis sp.
Stenopirates sp.
Stenopirates sp.PJ-2015
Nepomorpha
Enithares tibialis
Helotrephes sp.
Ilyocoris cimicoides
Laccotrephes robustus
Lethocerus deyrollei
Nerthra indica
Coleorrhyncha
Hackeriella veitchi
Hemiodoecus leai
Peloridium hammoniorum
Cicadomorpha
Abidama producta
Aeneolamia contigua
Alobaldia tobae
Aphrophora alni
Callitettix biformis
Callitettix braconoides
Callitettix sp
Callitettix versicolor
Empoasca vitis
Exitianus indicus
Homalodisca vitripennis
Illinigina sp
Leptobelus gazella
Leptobelus sp.
Meimuna opalifera
Norvellina sp.
Olidiana sp.
Orosius orientalis
Paphnutius ruficeps
Philaenus spumarius
Phlogotettix sp.
Platypleura kaempferi
Pomponia linearis
Tricentrus sp.
Typhlocyba sp.
Yanocephalus yanonis
Fulgoromorpha
Geisha distinctissima
Laodelphax striatella
Laodelphax striatellus
Laodelphax striatellus(2)
Laternaria candelaria
Lycorma delicatula
Nilaparvata lugens
Ricania marginalis
Sivaloka damnosus
Sogatella furcifera
Sternorrhyncha
Aleurocanthus spiniferus
Aphis craccivora
Aphis glycines
Aphis gossypii
Cavariella salicicola
Cervaphis quercus
Diuraphis noxia
Paratrioza sinica
Sitobion avenae
Superfamily level:
Lygaeoidea
Chauliops fallax
Kleidocerys resedae
Yemmalysus parallelus
Pentatomiodea
Acanthosoma labiduroides
Coptosoma bifaria
Dalcantha dilatata
Dolycoris baccarum
Eusthenes cupreus
Lamprocoris roylii
Megacopta cribraria
Nezara viridula
Sastragala edessoides
Urochela quadrinotata
Urostylis sp.
Family level:
Alydidae
Aradacanthia heissi
Brachyrhynchus hsiaoi
Neuroctenus parus
Riptortus pedestris
Miridae
Adelphocoris fasciaticollis
Apolygus lucorum
Lygus hesperus
Lygus lineolaris
Lygus rugulipennis
Nesidiocoris tenuis
Reduviidae
Agriosphodrus dohrni
Brontostoma colossus
Carcinocoris binghami
Cnizocoris sinensis
Macracanthopsis nodipes
Oncocephalus breviscutum
Peirates arcuatus
Phymata americana
Sirthenea flavipes
Sphedanolestes impressicollis
Triatoma dimidiata
Valentia hoffmanni
Nabidae
Alloeorhynchus bakeri
Gorpis annulatus
Gorpis humeralis
Himacerus apterus
Himacerus nodipes
Nabis apicalis
Aphididae
Aphis craccivora
Aphis glycines
Aphis gossypii
Cavariella salicicola
Diuraphis noxia
Sitobion avenae
Cercopidae
Abidama producta
Aeneolamia contigua
Aphrophora alni
Callitettix biformis
Callitettix braconoides
Callitettix sp
Callitettix versicolor
Paphnutius ruficeps
Cicadellidae
Alobaldia tobae
Empoasca vitis
Exitianus indicus
Homalodisca vitripennis
Illinigina sp.
Norvellina sp.
Olidiana sp.
Orosius orientalis
Phlogotettix sp.
Typhlocyba sp.
Yanocephalus yanonis
Cicadidae
Meimuna opalifera
Platypleura kaempferi
Pomponia linearis
Delphacidae
Laodelphax striatella
Laodelphax striatellus
Laodelphax striatellus
Nilaparvata lugens
Sogatella furcifera
